# Heterogeneity of HIV incidence: a comparative analysis between fishing communities and in a neighbouring rural general population, Uganda, and implications for HIV control

**DOI:** 10.1136/sextrans-2015-052179

**Published:** 2016-03-01

**Authors:** A Kamali, R N Nsubuga, E Ruzagira, U Bahemuka, G Asiki, M A Price, R Newton, P Kaleebu, P Fast

**Affiliations:** 1 Medical Research Council/Uganda Virus Research Council, Uganda Research Unit on AIDS, Entebbe, Uganda; 2 International AIDS Vaccine Initiative, New York, New York, USA; 3 Department of Health Sciences, University of York, York, UK; 4 London School of Hygiene and Tropical Medicine, London, UK

**Keywords:** HIV, AFRICA, EPIDEMIOLOGY (GENERAL)

## Abstract

**Objectives:**

To describe HIV heterogeneity in rural Uganda using incidence data collected between January 2012 and December 2014 among fishing cohort (FC) and in an adjacent rural general population cohort (GPC).

**Methods:**

In the FC, eligible HIV high-risk adults aged 18+ years were enrolled, followed and HIV tested every 3 months. Demographic and sexual behaviour data were also collected. The GPC, approximately 47 km away from the FC, was followed through annual surveys, and sociodemographic and behavioural data collected. A subset of GPC with comparable risk profiles to the FC was selected. We presented sociodemographic and risk profiles and also computed stratified HIV incidence. Cox regression was used to assess factors associated with HIV incidence.

**Results:**

Overall HIV incidence was higher in the FC than in the ‘high-risk’ GPC, 6.04 and 0.56 per 100 person years at risk, respectively, with a rate ratio (RR) of 10.83 (95% CI 6.11 to 19.76). This was higher among those aged 18–24 years, unmarried and those with more than two sex partners in the past year, RR of 15.44, 22.99 and 19.29, respectively. In the FC, factors associated with high incidence in multivariate analysis were duration in the community and unprotected sex. The factors in the GPC were ethnicity, marital status and duration in the community.

**Conclusions:**

We have observed a substantial heterogeneity in HIV incidence. The high incidence in fishing communities is contributing greatly to the overall HIV burden in Uganda, and thus urgent combination prevention efforts are needed towards national goal to reduce HIV epidemic.

## Introduction

After more than three decades, the spread of HIV continues, with approximately 2.1 million new infections globally in 2013, of which 1.6 million were in sub-Saharan Africa (SSA). This represents a 38% decline from 3.4 million in 2001, with a corresponding decline in AIDS deaths from 2.3 million in 2005 to 1.6 million in 2012.^1^ Though the HIV spread in SSA is predominantly through heterosexual transmission, studies reveal substantial between-country and within-country heterogeneity in HIV prevalence and incidence rates.^2^ ^3^ These differences have been attributed to the stage of the epidemic, sociodemographic, behavioural and biological factors. To what extent this heterogeneity exists within relatively close communities in countries with generalised HIV epidemics such as Uganda, has not been described.

From the late 1980s, several systems were set up in Uganda to monitor the epidemic. These included population-based epidemiological studies,^4^ ^5^ sentinel surveillance systems using antenatal and sexually transmitted disease clinic attendees and periodic national sero-behavioural surveys. These have provided valuable data for monitoring trends and for planning and policy formulation in Uganda and other African populations.

However, there has not been close monitoring of the epidemic among key populations. There is evidence that HIV prevalence in Uganda and in other countries may be elevated in key populations that include female sex workers, and residents of fishing communities.^6^ In Uganda, HIV was first reported in 1983 in a fishing village on the shores of Lake Victoria, spreading rapidly across the country, mainly along the trans-African and other major highways.^7^ It is estimated that HIV prevalence in African fishing communities is much higher (3–4 times) than the national average.^8–10^ HIV incidence in general populations is estimated at approximately 1 case per 100 person years of follow-up.^11–13^ The rates are much higher in fishing communities in general, with rates of 3.39 per 100 person years^14^ and among high-risk individuals within the same communities, with rates of 4.9 per 100 person years.^15^ Incidence rates are even higher in subgroups of the fishing communities such as younger persons (<30 years), those working in bars and among those who drink alcohol. The high rates have been attributed to a number of factors such as limited access to HIV prevention and treatment services, high alcohol consumption and behavioural characteristics including multiple and concurrent sexual partners as well as sexual networks.^10^ ^16–18^

In this study, we highlight heterogeneity of the HIV epidemic in rural Uganda using HIV incidence data collected between January 2012 and December 2014 among sexually active adults living in fishing and in an adjacent rural general population community, approximately 47 km apart. We discuss the implications for HIV prevention in such settings.

## Methods

### Study area and population

The fishing communities are located on the shores of Lake Victoria in Masaka District (South-West Uganda), approximately 38 km from the trans-African highway and with a population of approximately 6000 inhabitants. The major economic activities are fish related (63%), working in bars (11%) and other business (11%). There is also relatively high level of commercial sex work. The health services are low and provide limited primary healthcare. The fishing cohort was established in 2012, under International AIDS Vaccine Initiative Protocol B, as an HIV incidence open cohort to determine the feasibility of recruiting and following HIV uninfected participants at risk for HIV infection and assesses their suitability for phase II/III HIV prevention efficacy trials.^19^ One of the primary objectives is to estimate HIV incidence among at-risk volunteers. Since January 2012, about 564 adults (38% women) volunteers aged 18–49 years who met a predefined risk profile were enrolled.

**Figure 1:**
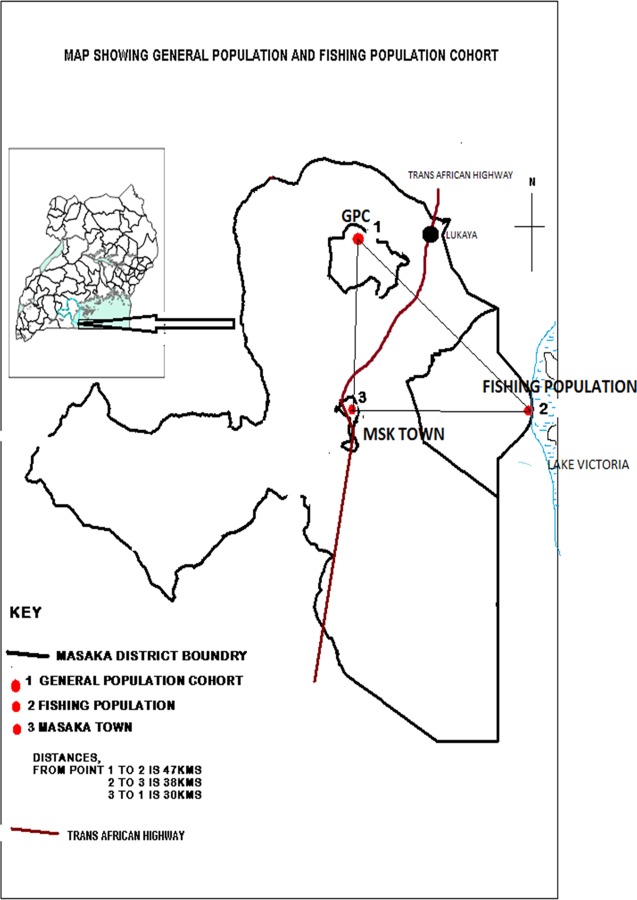
Map showing general population and fishing population cohort.

The general population cohort (GPC) was established in late 1989, initially including approximately 10 000 people residing in 15 neighbouring villages and later (1999) the cohort was expanded to an additional 10 villages with a population of approximately 8000 in order to increase accrual of HIV events and to allow more reliable estimates of HIV incidence and prevalence.^12^ The study area is a rural subcounty situated about 16 km from the trans-African highway and 47 km from the nearest fishing community (figure 1). Seasonal dirt roads and footpaths connect all the villages. The major economic activity of the GPC is peasant agriculture (68%) with food crops and cash crops (coffee).

### Study procedures

In the fishing cohort, basic study information was provided through community meetings and those who met basic eligibility criteria (age, residence and interest in study) were invited to attend the study clinic at Masaka town (38 km away) for detailed study information and screening. Eligible persons per protocol, defined as being sexually active and at risk for HIV infection were enrolled and followed every 3 months. HIV risk was defined by at least one of the following (in the past 3 months): current or past sexually transmitted infection (STI), unprotected sexual intercourse with more than one partner, unprotected sexual intercourse with new partner, being away from home for at least two nights in a month and/or weekly or more frequent alcohol use. At each visit, demographic, sexual behaviour and medical history data were collected from consenting individuals, and a complete physical examination and HIV counselling and testing (HCT) were performed. Venous blood was drawn for HIV and syphilis serology.

The GPC has been described in detail before but briefly the population has been followed annually through house-to-house demographic and serological surveys among resident adults aged 13 years and above after informed consent.^20^ Beginning in 2012, the house-to-house surveys approach was changed to biennial surveys conducted at a central hub in each village. This was done in order to improve cost-effectiveness of the survey, improve efficiency of data collection, increase population coverage and improve uptake of HIV test results. Participation rate using house-to-house survey approach was approximately 70%.^20^ Preliminary data indicate that participation at the hubs has increased to over 85%. At each survey, sociodemographic, behavioural and health-related data were collected, and a symptom-directed medical examination was done. The high-risk profile was defined by: reporting at least one sexual partner in the past year; unprotected sex at the most recent encounter or alcohol consumption in the past month.

All volunteers found to be HIV positive at the initial visit and those who sero-converted during follow-up received extra counselling and were referred to preferred HIV/AIDS treatment providers for enrolment into care. The research team provided free medical care for common ailments and referred those requiring specialised care to the national health providers.

Ethics approvals were by the Uganda Virus Research Institute Ethics Committee (GC/127/10/10/25) and the Uganda National Council for Science and Technology (HS870) for the fishing cohort, and GC/127/11/112/01 and MV834 for the GPC. All volunteers underwent an informed consent procedure and written informed consent documented.

### Statistical methods

By design, the fishing cohort was high risk for HIV acquisition. Analysis included participants enrolled and followed from 2012 to 2014. To achieve the purpose of this analysis, from the GPC, we defined and selected a high-risk subgroup of HIV-negative adults aged 18–49 years. The definition of high risk in the GPC was determined using data that were collected during surveys and these slightly varied from those fishing cohort. In order to have at least two HIV test results and also to accrue enough person years for HIV incidence calculation, we included high-risk individuals from 2010 to 2013 and followed them up to 2014. STI data were not collected in the GPC during the study period.

For both cohorts, we presented the distribution of the sociodemographic and risk profile factors and also computed stratified HIV incidence. We used Cox regression to assess factors associated with HIV incidence. We performed univariate and multivariate analysis; for the multivariate model, we included age and sex as potential confounders and any other variable with a univariate p<0.15 to avoid risk of excluding potential risk factors associated with HIV incidence. For multivariate analysis, the level of significance was 0.05. All analyses were performed using STATA V.12 (StataCorp, College Station, Texas, USA).

## Results

There were 564 adults enrolled in the fishing cohort of whom 87% were seen at two or more visits (table 1). The main reason for loss to follow-up was being away for work.

**Table 1:** Sociodemographic characteristics at baseline and HIV risk profile factors

Characteristic	Category	Fishing: no. enrolled with total visits ≥2 (%)	GPC: no. enrolled with total visits ≥2 (%)	p Value: comparison of proportions between cohorts
	All participants	490 (out of 564)		1213 (out of 1398)
Sex	Male	304 (62%)	956 (79%)	<0.001
	Female	186 (38%)	257 (21%)	<0.001
Age at enrolment	30+ years	185 (38%)	592 (49%)	0.009
	25–29 years	126 (26%)	200 (16%)	0.028
	18–24 years	179 (36%)	421 (35%)	0.815
Ethnicity	Muganda	214 (44%)	837 (69%)	<0.001
	Munyarwanda	107 (22%)	240 (20%)	0.671
	Other	169 (34%)	136 (11%)	<0.001
Marital status	Married	237 (49%)	745 (61%)	0.001
	Divorced/widowed	109 (22%)	14 (2%)	0.077
	Single	144 (29%)	454 (37%)	0.08
Occupation	Fish-related	309 (63%)	3 (0.25%)	0.026
	Bar/restaurant worker	53 (11%)	3 (0.25%)	0.553
	Business/trade	51 (10.4%)	99 (8%)	0.623
	Farming	5 (1%)	828 (68%)	0.001
	Formal/other	46 (8.3%)	176 (14.5%)	0.269
	Not earning	31 (6.3%)	104 (9%)	0.634
Duration in community	≥5 years	178 (36%)	800 (66%)	<0.001
	1–4 years	200 (41%)	351 (29%)	0.004
	≤1 year	112 (23%)	62 (5%)	0.002
*HIV risk profile factors*				
No. of sexual partners in the past year	Two or less	41 (8%)	1004 (83%)	<0.001
	More than two	449 (92%)	209 (17%)	<0.001
Unprotected sex in past 3 months or during last sex act	No	191 (39%)	332 (27%)	0.004
	Yes	299 (61%)	811 (67%)	0.062
	Information missing	–	70 (6%)	
Alcohol use in past month	No	140 (29%)	839 (69%)	<0.001
	Yes	350 (71%)	374 (31%)	<0.001
Current STI or STI in past 3 months	No	215 (44%)	–	
	Yes	275 (56%)	–	

GPC, general population cohort; STI, sexually transmitted infection.

In the GPC, a total of 1398 adults fitted the ‘high risk’ definition and 87% had data in two or more survey rounds. Similarly, the main reason for non-participation was absence of either visiting relatives or work related. There were differences in sociodemographic characteristics between the cohorts, explained partly by the migrant and mobile population in fishing communities attracted by fishing-related and sex work (females), and the lack of family and social structures. The high male proportion in the GPC is likely to be that males tend to report high-risk behaviours than females. As expected, the occupations in both populations were different with fishing cohort engaging mainly in fish-related activities and GPC mainly farming. The fishing cohort tended to have more recent immigrants (duration in community of less than 1 year) than the GPC (23% vs 5%).

The HIV risk factors differed between the two cohorts. The fishing cohort had over 90% of participants reporting more than two partners in past year compared with 17% in the GPC (p<0.001), and higher alcohol consumption, 71% vs 31% (p<0.001). There was no difference in history of unprotected sex in the past 3 months or latest sexual encounter (p=0.062). The fishing cohort had 56% of participants reporting current or history of an STI (no data were available in the GPC).

### HIV incidence

There were a total of 36 HIV incident cases over 596.1 person years at risk (PYAR) in the fishing cohort with an incidence rate of 6.04 per 100 PYAR (95% CI 4.36 to 8.37). In the ‘high-risk’ GPC, a total of 20 HIV incident cases were identified during 3590 PYAR with an incidence rate of 0.56 (95% CI 0.36 to 0.86) per 100 PYAR. Overall, the HIV incidence was higher in the fishing cohort than among the ‘high-risk’ GPC participants with a rate ratio (RR) of 10.83 (95% CI 6.11 to 19.76). This was even higher within certain demographic and risk profiles such as young age (18–24 years), unmarried and among those with more than two sex partners in the past year (table 2). In the fishing cohort, incidence was higher in females than in males, 9.29 (95% CI 5.93 to 14.56) and 4.34 (95% CI 2.70 to 6.98), respectively. The factors associated with high HIV incidence in the univariate analysis were sex (being female), duration in community (less than 1 year), occupation (working in a bar), marital status (divorced/widowed), engaging in unprotected sex and history of STI. However, only duration in the community and engaging in unprotected sex remained significant in multivariate analysis. Those who had lived in the area for less than a year had an adjusted RR of 7.64 (95% CI 2.36 to 24.67) compared with participants who had lived in the area for at least 5 years. Participants who reported engaging in unprotected sex were three times more likely to acquire HIV infection compared with those who did not report having unprotected sex; RR of 3.11 (95% CI 1.27 to 7.66). History of STI was borderline significant.

**Table 2:** Crude and multivariate analysis of HIV incidence associated factors and comparison of incidence rates between the cohorts

		Fisher folk cohort						General population cohort							
		HIV incident			Crude analysis	Multivariate analysis		HIV incident			Crude analysis	Multivariate analysis		Comparison of incidence rates between the cohorts	
Characteristic	Category	Cases	PYAR	RR (95% CI)/100	RR (95% CI)	RR (95% CI)	p Value	Cases	PYAR	RR (95% CI)/100	RR (95% CI)	RR (95% CI)	p Value	RR (95% CI)/100	p Value
	All participants	36	596.1	6.04 (4.36 to 8.37)				20	3589.6	0.56 (0.36 to 0.86)				10.83 (6.11 to 19.76)	< 0.001
Sex	Male	17	391.5	4.34 (2.70 to 6.98)	Ref	Ref	0.73	14	2823.8	0.50 (0.29 to 0.84)	Ref	Ref	0.88	8.76 (4.06 to 19.19)	<0.001
	Female	19	204.5	9.29 (5.93 to 14.56)	1.97 (1.03 to 3.76)	1.20 (0.39 to 3.63)		6	765.8	0.78 (0.35 to 1.74)	1.58 (0.61 to 4.10)	0.92 (0.32 to 2.69)		11.86 (4.55 to 36.28)	<0.001
Age at enrolment	30+ years	14	236.0	5.93 (3.51 to 10.02)	Ref	Ref	0.51	10	1821.7	0.55 (0.30 to 1.02)	Ref	Ref	0.42	10.81 (4.46 to 27.19)	<0.001
	25–29 years	9	156.5	5.75 (2.99 to 11.05)	0.96 (0.42 to 2.22)	0.69 (0.30 to 1.59)		5	559.5	0.89 (0.37 to 2.15)	1.63 (0.56 to 4.73)	1.84 (0.70 to 4.84)		6.43 (1.94 to 24.44)	0.001
	18–24 years	13	203.5	6.39 (3.71 to 11.00)	1.03 (0.48 to 2.21)	0.60 (0.22 to 1.63)		5	1208.4	0.41 (0.17 to 0.99)	0.75 (0.26 to 2.18)	2.08 (0.39 to 10.98)		15.44 (5.16 to 55.31)	<0.001
Ethnicity	Muganda	16	257.3	6.22 (3.81 to 10.15)	Ref			10	2481.4	0.40 (0.22 to 0.75)	Ref	Ref	**0.05**	15.43 (6.58 to 38.04)	<0.001
	Munyarwanda	6	132.1	4.54 (2.04 to 10.11)	0.73 (0.29 to 1.87)			9	713.0	1.26 (0.66 to 2.43)	3.14 (1.28 to 7.73)	2.84 (1.18 to 6.83)		3.60 (1.05 to 11.32)	0.025
	Other	14	206.7	6.77 (4.01 to 11.44)	1.09 (0.54 to 2.23)			1	395.1	0.25 (0.04 to 1.80)	0.63 (0.08 to 4.91)	0.59 (0.07 to 4.76)		26.76 (4.07 to 1131)	<0.001
Marital status	Married	15	296.1	5.07 (3.05 to 8.40)	Ref	Ref	0.2	18	2235.2	0.81 (0.51 to 1.28)	Ref	Ref	**0.003**	6.29 (2.95 to 13.22)	<0.001
	Divorced/widowed	15	127.5	11.76 (7.09 to 19.51)	2.23 (1.09 to 4.57)	1.17 (0.49 to 2.75)		0	33.0	0	–	–		–	
	Single	6	172.4	3.48 (1.56 to 7.75)	0.68 (0.27 to 1.75)	0.49 (0.17 to 1.42)		2	1321.4	0.15 (0.04 to 0.61)	0.19 (0.04 to 0.81)	0.10 (0.01 to 0.76)		22.99 (4.11 to 233)	<0.001
Occupation	Fish-related	19	394.8	4.81 (3.07 to 7.55)	Ref	Ref	0.45	0	8.2	0	–			–	
	Bar/restaurant/hotel worker	11	54.4	20.24 (11.21 to 36.54)	3.80 (1.82 to 7.96)	1.83 (0.63 to 5.29)		0	6.5	0	–			–	
	Farming	0	7.8	0	–	–		14	2533.3	0.55 (0.33 to 0.93)	Ref			–	
	Business/trade	3	59.2	5.07 (1.63 to 15.71)	0.99 (0.30 to 3.31)	1.01 (0.26 to 3.88)		2	293.2	0.68 (0.17 to 2.73)	1.21 (0.28 to 5.29)			7.43 (0.85 to 88.96)	0.04
	Formal/other	2	45.2	4.42 (1.11 to 17.67)	0.85 (0.20 to 3.63)	0.64 (0.13 to 3.07)		3	449.8	0.67 (0.21 to 2.07)	1.18 (0.34 to 4.07)			6.63 (0.55 to 57.91)	0.076
	Un employed	1	34.7	2.88 (0.41 to 20.44)	0.57 (0.07 to 4.33)	0.42 (0.04 to 4.05)		1	298.6	0.33 (0.05 to 2.38)	0.60 (0.08 to 4.52)			8.60 (0.11 to 675)	0.208
Duration in community	≥5 years	5	245.2	2.04 (0.85 to 4.90)	Ref	Ref	**<0.001**	11	2484.2	0.44 (0.25 to 0.80)	Ref	Ref	**0.04**	4.61 (1.25 to 14.38)	0.013
	1–4 years	13	234.3	5.55 (3.22 to 9.56)	2.59 (0.92 to 7.31)	2.88 (0.95 to 8.78)		5	945.2	0.53 (0.22 to 1.27)	1.18 (0.41 to 3.38)	1.60 (0.53 to 4.89)		10.49 (3.51 to 37.58)	<0.001
	≤1 year	18	116.6	15.43 (9.72 to 24.5)	6.87 (2.54 to 18.58)	7.64 (2.36 to 24.67)		4	160.2	2.50 (0.94 to 6.65)	5.43 (1.72 to 17.10)	5.98 (2.02 to 17.72)		6.18 (2.04 to 25.11)	<0.001
*HIV risk profile factors*															
Number of sexual partners in the past year	Two or less	1	47.4	2.11 (0.30 to 14.98)	Ref			18	2984.9	0.60 (0.38 to 0.96)	Ref			3.50 (0.08 to 22.16)	0.294
	More than two	35	548.7	6.38 (4.58 to 8.88)	3.09 (0.42 to 22.67)			2	604.7	0.33 (0.08 to 1.32)	0.54 (0.13 to 2.33)			19.29 (4.95 to 165)	<0.001
Had unprotected sex in past 3 months/year	No	8	219	3.65 (1.83 to 7.31)	Ref	Ref	**0.006**	7	928.8	0.75 (0.36 to 1.58)	Ref		** **	4.85 (1.53 to 15.7)	0.004
	Yes	28	377.1	7.43 (5.13 to 10.75)	2.13 (0.97 to 4.66)	3.11 (1.27 to 7.66)		13	2472.5	0.53 (0.31 to 0.91)	0.70 (0.28 to 1.73)			14.12 (7.08 to 29.7)	<0.001
	Information missing	–	–	–	–	–		0	188.3	0	–				
Alcohol use in past month	No	8	151.9	5.27 (2.63 to 10.53)	Ref			12	2499.6	0.48 (0.27 to 0.85)	Ref			10.97 (3.89 to 29.18)	<0.001
	Yes	28	444.2	6.30 (4.35 to 9.13)	1.26 (0.57 to 2.77)			8	1090	0.73 (0.37 to 1.47)	1.50 (0.61 to 3.67)			8.59 (3.81 to 21.81)	<0.001
Current STI or STI in past 3 months	No	11	272.2	4.04 (2.24 to 7.30)	Ref	Ref	**0.08**	–	–	–	–	–	–		
	Yes	25	323.9	7.72 (5.22 to 11.42)	1.85 (0.91 to 3.76)	1.89 (0.90 to 4.00)		–	–	–	–	–	–		

PYAR, person years at risk; RR, rate ratio; STI, sexually transmitted infection.

In the ‘high-risk’ GPC, variables univariately associated with HIV incidence (ethnicity, marital status and duration in community) remained significantly associated with HIV incidence in multivariable model. Participants of Rwandan origin were more at risk of HIV infection compared with the native Baganda with an incidence RR of 2.84 (95% CI 1.18 to 6.83); the singles were at lower risk of infection compared with the married, RR 0.10 (95% CI 0.01 to 0.76); while those with less than 1 year of residence in the community were more likely to be infected, RR 5.98 (95% CI 2.02 to 17.72) compared with the most stable residents. There was no difference in incidence rates by reported number of sexual partners, unprotected sex or alcohol consumption.

## Discussion

This work has examined HIV incidence in two geographical close cohorts that have been under surveillance. We have observed a substantial heterogeneity in HIV incidence; 11 times higher in the fishing cohort than among the ‘high-risk’ GPC and more marked within certain demographic and risk profiles. These cohorts, however, differ in composition: one being a fishing population and the other predominantly farming general population. The fishing cohort is high risk by design and we selected a ‘high-risk’ subpopulation from the GPC using known HIV risk factors. We have also observed differences in reported sexual partners (over 90% participants reporting more than two partners among the fishing cohort; and over 80% reporting two or less partners in past year in GPC), but similar in reported condom use. It is important to note other differences such as the high level of commercial sex and low availability of HIV prevention and treatment services in the fishing cohort) could also account for the differences in HIV incidence. In the ‘high-risk’ GPC, the overall HIV incidence within the different demographic and risk profiles was low. Using known HIV risk factors in generalised HIV epidemic within rural settings may therefore not be sufficient enough to characterise risk and prioritise interventions.

We have previously described HIV incidence in fishing communities showing high infection rates^15^ ^16^ and in the GPC.^12^ ^21^ ^22^ Other studies have described similar HIV infection rates in fishing communities.^10^ ^14^ The high HIV rates in fishing communities have been attributed to several factors such as mobility, level of sex work and relatively young population with a daily income to spend on sex and alcohol, and inadequate health services.

Heterogeneity in HIV infection rates has also been described at global level,^23^ across Africa^24^ and within individual countries.^25^ Heterogeneity at global level can be attributed to differences in economic development and better health services in developed countries, whereas in Africa, factors such as high rates of sexual mixing and STIs, and low condom use account for higher HIV infection. Heterogeneity in some African countries has also been related to proximity to primary and secondary roads.^2^ ^26^ The likely explanations for the differences in HIV incidence in our cohorts are the differences in sociodemographic characteristics such as higher proportion of young adults, rates of divorced/widowed, mobility and alcohol consumption in fishing cohort. The access to health services and socioeconomic inequalities could also partly be contributing to these differences.

Differences in STI prevalence is another contributing factor to the heterogeneity in these two populations. We have previously reported prevalence of bacterial STIs and reported urethral and vaginal discharge in the GPC.^27^ Gonorrhoea and chlamydia prevalence was approximately 1% and reported urethral and vaginal discharge was 3% and 10%, respectively. The STI prevalence reported among the fishing cohort was 50% or more.

We acknowledge that there may be limitations to our study and these include: First, the classification of ‘high-risk’ individuals was based on reported sexual behaviour which is sometimes unreliable and may be over-reported or under-reported. Our research teams have good experience in collecting such data and had built good rapport with the respondents over a long period. There was therefore less discomfort and difficulty in responding to sexual behaviour questions. Our findings particularly in the fishing cohort also indicate a good correlation between HIV incidence and high-risk sexual behaviour and alcohol consumption. Second, the two studies were not designed to be compared and as such variable definitions, such as definition of high risk did not match each other perfectly in particular data on history of STIs. Third, we were not able to obtain data on rates of male circumcision (MC) in both cohorts to assess its effect on HIV incidence. National average of MC in this region is approximately 20%–30%, majority of whom are of Islam faith and there have not been any roll out circumcision in both populations; so this is not likely to fully explain the difference. Fourth, the HIV antiretroviral treatment (ART) programme was initiated in 2004 in the GPC and to date the coverage is approximately 50%. We do not know the coverage in fishing communities and considering the geographical isolation of fishing communities coupled with mobility and access, the ART coverage is likely to be lower. This difference could partly explain the higher HIV transmission rates in fishing due to a higher community viral load.

In summary, this study has demonstrated a huge variability in HIV incidence between high-risk adults living in a fishing community and in a subset of high-risk residents in an adjacent GPC. The exact size of the population engaged in fishing activities in Uganda is estimated at up to three million people.^28^ There are very limited healthcare and other basic services in fishing communities, with communities difficult to access such as isolated islands.^29^ At the same time, HCT has only been provided through our research team. Thus, health facility-based HIV interventions such as health education, HCT and STI treatment are very limited. The high incidence we and others have observed implies that these communities contribute a great proportion to the overall burden of HIV in Uganda, and thus urgent HIV combination prevention efforts, treatment and care are needed in such communities towards national goal to reduce HIV epidemic. These efforts should be well tailored and above the routine national HIV prevention programmes.

**Key messages**

- HIV incidence is approximately 11 times higher in fishing cohort than in a subpopulation living in an adjacent rural community, and is more marked within some demographic and risk profiles.
- Observed HIV incidence in fishing communities is contributing greatly to the overall HIV burden in Uganda, and possibly in other similar settings.
- Urgent HIV combination prevention efforts, including treatment and care are needed in such communities towards national goal to reduce HIV epidemic.
